# GSPE Inhibits HMGB1 Release, Attenuating Renal IR-Induced Acute Renal Injury and Chronic Renal Fibrosis

**DOI:** 10.3390/ijms17101647

**Published:** 2016-09-29

**Authors:** Juan Zhan, Kun Wang, Conghui Zhang, Chunxiu Zhang, Yueqiang Li, Ying Zhang, Xiaoyan Chang, Qiaodan Zhou, Ying Yao, Yanyan Liu, Gang Xu

**Affiliations:** Department of Nephrology, Tongji Hospital, Tongji Medical College, Huazhong University of Science and Technology, 1095 Jiefang Ave, Wuhan 430030, Hubei, China; zhan021989juan@foxmail.com (J.Z.); wangkun901109@163.com (K.W.); yaoyaoyouxin@outlook.com (Co.Z.); zhangchunxiu03@163.com (Ch.Z.); wuanliyue@126.com (Yu.L.); zhangying19880914@163.com (Y.Z.); yanloveiy666@163.com (X.C.); qiaodanzhou_1000@163.com (Q.Z.); yaoyingkk@126.com (Y.Y.)

**Keywords:** GSPE, acute kidney injury, chronic kidney fibrosis, HMGB1

## Abstract

Grape seed proanthocyanindin extract (GSPE) is a polyphenolic bioflavonoid derived from grape seeds and has been widely studied for its potent antioxidant, anti-inflammatory and antitumor activities. HMGB1 is a newly discovered danger-associated molecular pattern (DAMP) that has potent proinflammatory effects once released by necrotic cells. However, the effect of GSPE on the HMGB1, and the relationship of those two with acute kidney injury and chronic kidney fibrosis are unknown. This study aimed to investigate the impact of GSPE on acute kidney injury and chronic fibrosis. C57bl/6 mice were subjected to bilateral ischemia/reperfusion (I/R) and unilateral I/R with or without GSPE administration. After bilateral I/R, mice administered GSPE had a marked improvement in renal function (BUN and Cr), decreased pathological damage and reduced inflammation. In unilateral I/R, mice subjected GSPE showed reduced tubulointerstitial fibrosis and decreased inflammatory reaction. The renoprotection of GSPE on both models was associated with the inhibition of HMGB1 nucleocytoplasmic shuttling and release, which can amplify the inflammation through binding to its downstream receptor TLR4 and facilitated P65 transcription. Thus, we have reason to believe that GSPE could be a good alternative therapy for the prevention and treatment of IR-induced renal injury and fibrosis in clinical practice.

## 1. Introduction

Acute kidney injury (AKI) is an important clinical syndrome associated with high morbidity and mortality [1]. Recent studies have shown that AKI is an important risk factor for the development of chronic kidney disease (CKD) [2,3]. As a major cause of AKI, renal ischemia reperfusion injury (IRI) is characterized by vascular dysfunction, immune system activation, and tubular epithelial cell injury [4,5]. With persistent tubulointerstitial inflammation and the cytokines released by the injured tubular epithelium after I/R [6,7], fibroblasts activate and secrete extracellular matrix (ECM) [8,9], which leads to the loss of kidney function and to renal fibrosis.

High-mobility group box 1 (HMGB1) is a DNA-binding protein that also acts as a damage-associated molecular pattern (DAMP) molecule [10]. It can be either passively released from necrotic cells or actively secreted by activated immune cells into the extracellular milieu [11,12], both of which then activates proinflammatory signaling pathways by interacting with Toll-like receptor 4 (TLR4), TLR2, and the receptor for advanced glycation end products (RAGE) [13,14]. Recent evidence has shown that HMGB1 can not only mediate injury and inflammation in acute renal IRI [15], but also participate in the release of TGF-β, the most potent cytokine promoting renal fibrosis, by tubular epithelial cells [16,17]. Inhibiting HMGB1 can significantly protect the kidney from both IR-induced acute damage and cyclosporine-induced chronic fibrosis [15,18]. Therefore, a therapeutic tool that prevents HMGB1 release from necrotic renal parenchyma cells and activated macrophages may have a potent protective effect on IR-induced acute renal injury and the subsequent fibrosis.

Grape seed proanthocyanindin extract (GSPE) is a type of polyphenolic bioflavonoid derived from whole grape seeds. It has been reported to exhibit a variety of properties, such as antioxidant, anti-inflammatory and antitumor activities [19,20,21]. Among these, we are mainly interested in its anti-inflammatory properties because inflammation is the key correlation between AKI and CKD. Administration of GSPE in mice is able to alleviate both acute renal injury induced by I/R and chronic renal fibrosis caused by diabetic nephropathy (DN) [22,23]. However, these previous studies ignored the inflammation and did not take into account the association between renoprotective effect of GSPE and HMGB1. Therefore, we hypothesized that GSPEs can alleviate IR-induced renal damage and long-term fibrosis by blocking the nucleocytoplasmic translocation and release of HMGB1, thereby preventing subsequent inflammatory responses. To test this hypothesis, we administered GSPEs to mice with moderate reversible bilateral I/R and unilateral I/R.

## 2. Results

### 2.1. Administration of GSPE Protects Mice against Bilateral IR-Induced AKI

**Figure 1:**
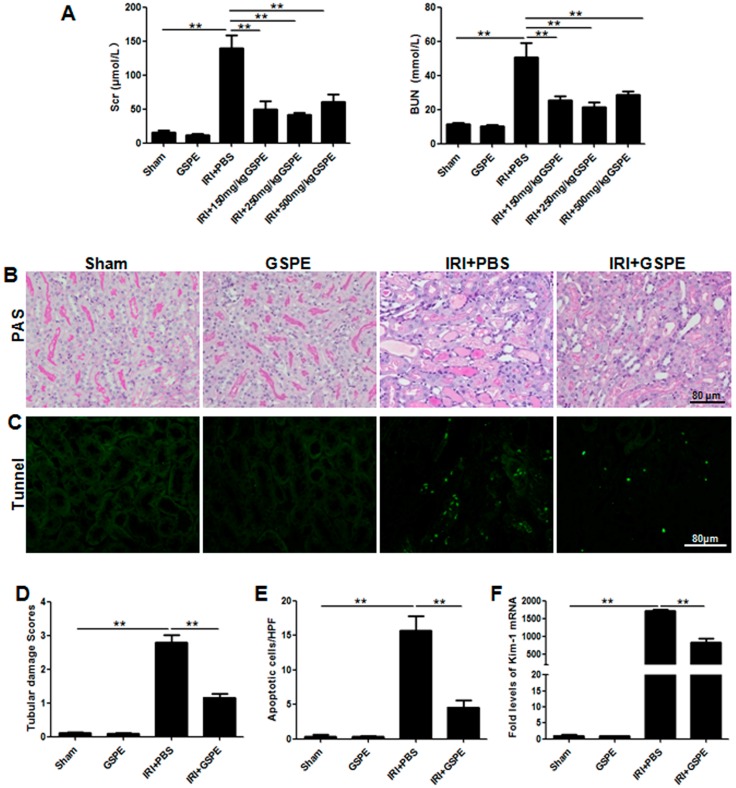
Administration of GSPE (grape seed proanthocyanindin extract) protects mice against bilateral IR-induced AKI (acute kidney injury). Mice pretreated with PBS or GSPE following bilateral IR or a sham operation served as controls. (**A**) Serum creatinine (Cr) and blood urea nitrogen (BUN) levels of the six groups; (**B**) PAS staining, showing pathological changes in each group; (**C**) Apoptosis in the kidney was assessed by the TUNEL assay; (**D**) Semi-quantitative analysis of PAS staining for the severity of renal injury; (**E**) Semi-quantitative analysis of TUNEL-positive cells; (**F**) Real-time PCR (Polymerase Chain Reaction) was used to measure mRNA expression of KIM-1. The data shown are mean ± SEM. (** *p* < 0.01; *n* = 7 per group).

We first sought to assess the impact of GSPE on renal function after bilateral IR-induced acute renal injury. For this purpose, mice were treated with GSPE for 5 days consecutively at three different doses, followed by bilateral IR insult as described, and blood was collected after 24 h of reperfusion. Mice from the GSPE-only group displayed similar levels of blood urea nitrogen (BUN) and serum creatinine (Cr) (Figure 1A) to those of mice from the sham group, indicating that GSPE did not cause a perceptible change in renal function. Compared with mice from the sham and GSPE-only group, mice from the PBS group (IRI+PBS) exhibited significantly increased serum Cr and BUN levels (Figure 1A). By contrast, pretreatment with GSPE preserved renal function, as proven by the significantly lower level of serum Cr and BUN (Figure 1A), and GSPE at a concentration of 250 mg/kg exhibited the most potent renoprotection; therefore, we choose this concentration for the following experiments.

To further confirm these observations, we conducted a histological analysis of renal sections. PAS staining revealed that the IRI+PBS group had widespread tubular necrosis, loss of the brush border, cast formation, and tubular dilatation at the site of the corticomedullary junction (Figure 1B,D), but all of those were markedly attenuated in GSPE-pretreated mice. The KIM-1 level correlated with the morphology described above (Figure 1F).

Next, we examined the effect of GSPE on IR-induced tubular apoptosis by a TUNEL assay. Renal cells derived from the kidneys of the IRI+PBS group exhibited severe apoptosis after 24 h of I/R (Figure 1C,E), while mice from IRI+GSPE group manifested a marked reduction in TUNEL-positive cells (Figure 1C,E).

### 2.2. Pre-Treatment with GSPE Attenuates the IR-Induced Inflammatory Reaction

**Figure 2:**
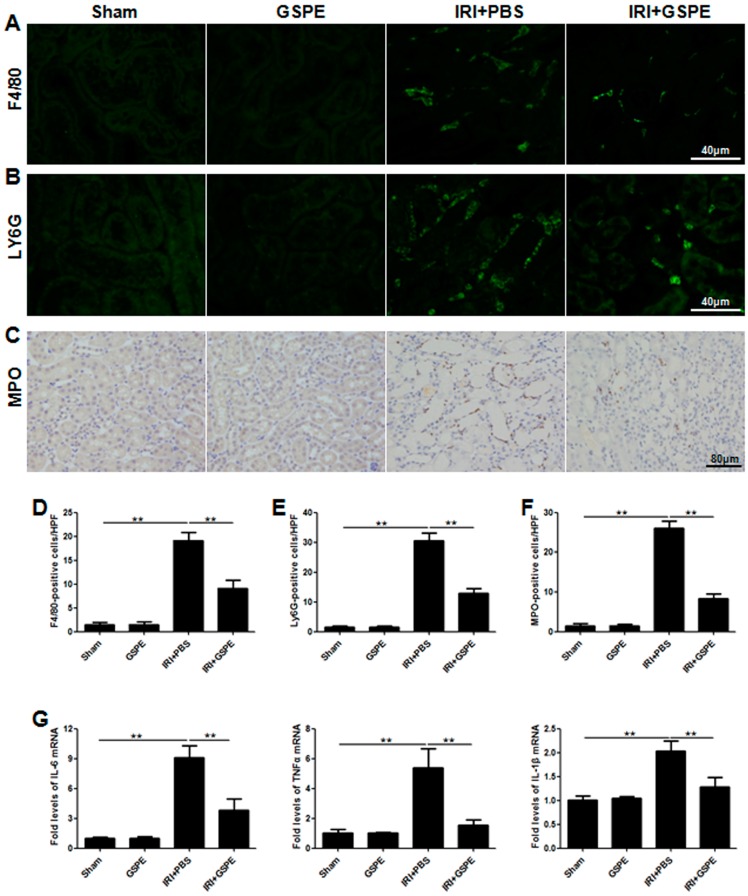
Pretreatment with GSPE attenuates the IR-induced inflammatory reaction. (**A**) Immunofluorescence staining of F4/80 was performed to analyze the infiltration of macrophages; (**B**) Ly6G staining was performed to assess the number of neutrophils; (**C**) Immunohistochemistry staining of MPO was performed to calculate the neutrophil infiltration; (**D**–**F**) Semi-quantitative assessment of F4/80, Ly6G and MPO staining; (**G**) Real-time PCR of IL-6, TNF-α and IL-1β was performed to evaluate the expression level of inflammatory cytokines in the kidney. The data shown are mean ±SEM (** *p* < 0.01; *n* = 7 per group).

To investigate whether the alleviated IR-induced AKI from the IRI+GSPE group was due to an attenuated inflammatory response, we then measured the infiltration of neutrophils (MPO +/Ly6G + cells) and macrophages (F4/80 + cells) in the tubulointerstitial region. As expected, in contrast to the negative staining in the sham and GSPE groups, mice subjected to IR insult had significant inflammatory cell infiltration, especially neutrophils (Figure 2A–F), while this reaction was markedly attenuated in the IRI+GSPE group (Figure 2A–F).

To further address the anti-inflammation effect of GSPE, pro-inflammatory cytokines were detected by real-time PCR. The IRI+PBS group showed strong upregulation of tumor necrosis factor-α (TNF-α), interleukin-6 (IL-6), and IL-1β (Figure 2G). By contrast, pre-treatment with GSPE markedly limited this increase (Figure 2G).

### 2.3. GSPE Pretreatment Inhibits the Release of Chemokines

**Figure 3:**
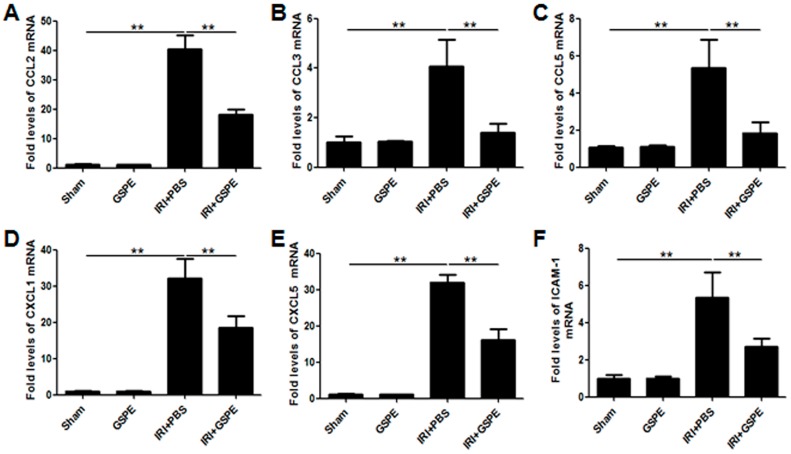
GSPE pretreatment inhibits the release of chemokines. The amplification of inflammation was measured by the mRNA expression of CCL2 (**A**), CCL3 (**B**), CCL5 (**C**), CXCL1 (**D**), CXCL5 (**E**) and ICAM-1 (**F**). The data shown are mean ±SEM (** *p* < 0.01; *n* = 7 per group).

As chemokines amplify inflammation, we next used real-time PCR to measure the mRNA expression of several important chemokines in the kidneys 24 h after reperfusion. Compared with the sham and GSPE-only groups, PBS-treated IR animals displayed a strong increase in expression of chemokine ligand 2 (CCL2) (Figure 3A), CCL3 (Figure 3B), CCL5 (Figure 3C), CXCL1 (Figure 3D), CXCL5 (Figure 3E) and intercellular adhesion molecule 1 (ICAM-1) (Figure 3F); however, all of these increases can be modulated by GSPE (Figure 3A–F).

### 2.4. Pretreatment with GSPE Suppresses HMGB1-TLR4-p65 Activity in AKI

**Figure 4:**
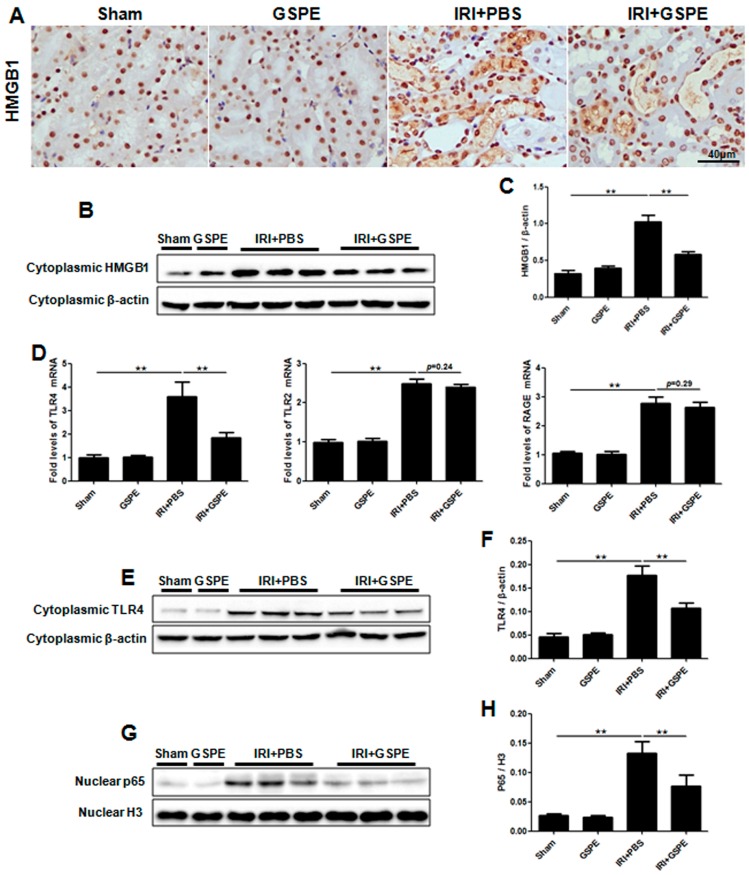
Pretreatment with GSPE suppresses HMGB1-TLR4-p65 activity in AKI. (**A**) Immunohistochemical staining of HMGB1 from sections of kidneys harvested from each of four groups; (**B**) Western blot analysis for HMGB1 was performed using cytoplasmic protein samples; (**C**) The bar graph shows the corresponding densitometry of HMGB1; (**D**) Expression levels of TLR4, TLR2 and RAGE in the kidney were detected by RT-PCR; (**E**) Representative result for Western blot analysis of cytoplasmic TLR4; (**F**) The relative expression of TLR4 is displayed by a histogram; (**G**,**H**) The expression of nuclear p65 was detected by Western blot, and the histogram shows the relative gray density of P65 corrected for histone 3. The data shown are mean ±SEM (** *p* < 0.01; *n* = 7 per group).

To gain insight into the underlying mechanisms by which GSPE inhibits the inflammatory response after IR insult, we first examined the expression of HMGB1, a potent trigger of inflammation. Immunohistochemical staining showed that in the kidneys of the sham and GSPE groups, HMGB1 was observed to be predominantly located in the nucleus of renal parenchyma cells, especially the renal tubular epithelial cells (Figure 4A). After reperfusion, a rapid increase in cytoplasmic HMGB1 was observed (Figure 4A), but only a minimal amount of HMGB1 could be detected after treatment with GSPE (Figure 4A). Western blot analysis of renal cytoplasmic proteins further confirmed the changes in HMGB1 passive release as described above (Figure 4B,C).

As extracellular HMGB1 activates proinflammatory signaling pathways by activating TLR4, TLR2 and RAGE [13,14], we measured the mRNA expression of these HMGB1 receptors in the kidneys by real-time PCR. IR insult induced upregulation of TLR4, but not TLR2 or RAGE (Figure 4D), and as before, all of these changes were attenuated by GSPE (Figure 4D). The cytoplasmic protein expression diversity of TLR4 was the same as that of mRNA (Figure 4E,F).

Given that p65 is the downstream effector protein of HMGB1-TLR4 signaling, we further investigated p65 activation by analyzing nuclear p65. As expected, administration of GSPE significantly inhibited p65 activation (Figure 4G,H).

### 2.5. GSPE Treatment Lessens Unilateral IR-Induced Chronic Renal Fibrosis

**Figure 5:**
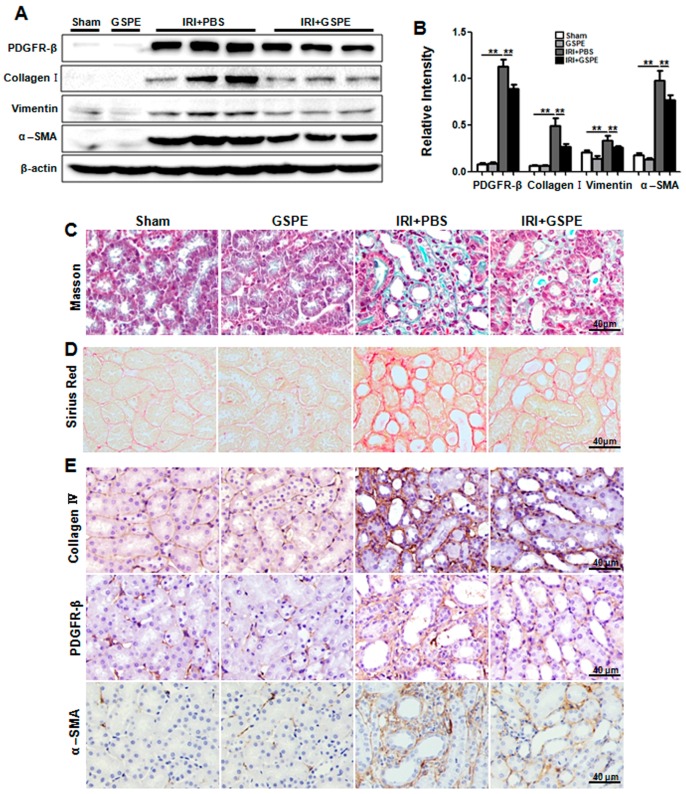
GSPE treatment lessens the unilateral IR-induced chronic renal fibrosis. Mice were treated with GSPE or PBS after exposure to unilateral IR for 30 min, and the kidneys were collected on the 14th day. (**A**) Western blot analysis of fibrotic markers induced by unilateral IR: PDGFR-β, collagen I, vimentin and α-SMA; (**B**) The histogram shows relative intensity for each marker normalized to β-actin. Masson’s (**C**) and Sirius Red (**D**) staining showing collagen deposition; (**E**) Immunohistochemical staining of fibrotic markers: PDGFR-β, collagen IV and α-SMA was performed to detect the degree of tubulointerstitial fibrosis in these four groups. The data shown are mean ±SEM (** *p* < 0.01; *n* = 7 per group).

Considering the potent anti-inflammatory effects of GSPE in AKI, we hypothesized that GSPE may also protect the kidneys from chronic fibrosis. Thus, we then administered GSPE for 14 days in succession to mice after they suffered from a unilateral IR insult. Surprisingly, Masson’s and Sirius Red staining showed much less severe interstitial fibrosis in GSPE-treated IR mice than in PBS-treated IR mice (Figure 5C,D). Western blot analysis also indicated lower levels of expression of fibrogenic markers PDGFR-β, collagen I, vimentin and α-SMA (Figure 5A,B) in mice from the IRI+GSPE group. Immunohistochemical staining of PDGFR-β, collagen IV, and α-SMA showed the same results as above (Figure 5E). All of these demonstrate that GSPE is a potent inhibitor of renal fibrosis.

### 2.6. Administration of GSPE Attenuates Unilateral IR-Induced Chronic Kidney Injury and Tubulointerstitial Inflammation

**Figure 6:**
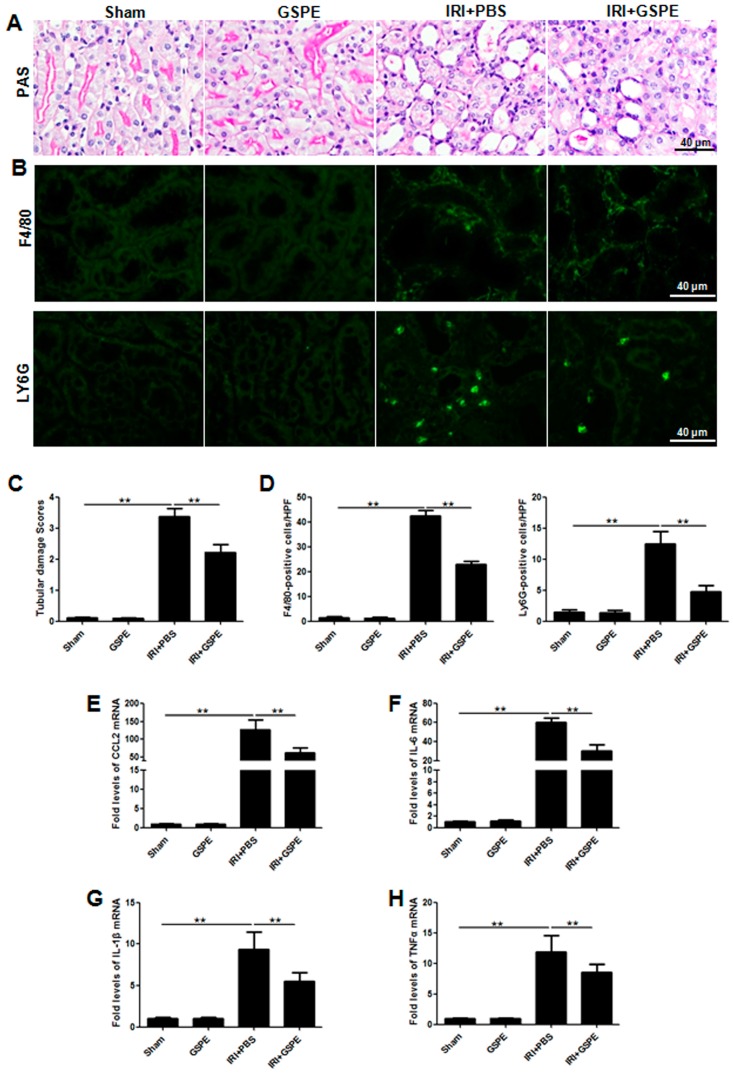
Administration of GSPE attenuates unilateral IR-induced chronic kidney injury and tubulointerstitial inflammation. (**A**) Morphological changes in kidney sections were examined by PAS staining; (**B**) Immunofluorescence staining of F4/80 and LY6G in the kidney; (**C**) Semi-quantitative analysis of PAS to assess tubular damage for all mice studied; (**D**) Semi-quantitative analysis of immunofluorescence staining of F4/80 and LY6G in the kidney, based on the number of positive cells in 10 random areas for all mice collected; (**E**–**H**) The expression of inflammatory cytokines CCL2 (**E**), IL-6 (**F**), IL-1β (**G**) and TNF-α (**H**) was measured by real-time PCR. The data shown are mean ±SEM (** *p* < 0.01; *n* = 7 per group).

The observations from unilateral IR mice prompted us to check the inflammation in the tubulointerstitial region. Notably, in contrast to mice from the unilateral IRI group, mice treated with GSPE can not only limit the infiltration of neutrophils (Ly6G + cells) and macrophages (F4/80 + cells) (Figure 5B,D) but also attenuate the increase in potent proinflammatory cytokines CCL2 (Figure 6E), IL-6 (Figure 6F), IL-1β (Figure 6G) and TNF-α (Figure 6H). As a result of reduced inflammation, the tubular brush border and the structure of tubular epithelia cells were preserved the best in the IRI+GSPE group (Figure 6A,C).

### 2.7. Successive Treatment with GSPE Suppresses the Activation of HMGB-TLR4-P65-TGF-β

**Figure 7:**
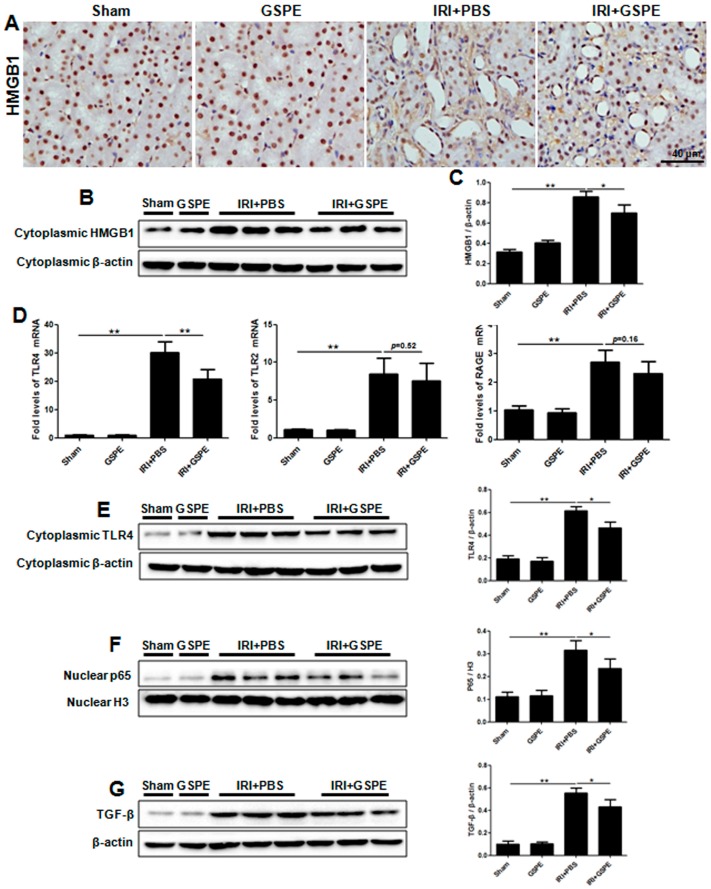
Successive treatment with GSPE suppresses the activation of HMGB-TLR4-p65-TGF-β. (**A**) Immunohistochemical staining showed HMGB1 released into the cytoplasm and the renal interstitium after the I/R operation; (**B**) Cytoplasmic HMGB1 was assessed by Western blot; (**C**) The gray intensity analyses for HMGB1 are shown in bar graphs; (**D**) Real-time PCR was employed to detect the mRNA level of TLR4, TLR2 and RAGE in the kidney; (**E**) The protein level of TLR4 in the renal cytoplasm was detected by Western blot; (**F**) Western blot analysis for P65 was performed using a nuclear protein sample; (**G**) Western blot analysis for TGF-β was performed using total protein samples from kidneys. The data shown are mean ±SEM (* *p* < 0.05, ** *p* < 0.01; *n* = 7 per group).

On the basis of research performed on AKI, we investigated the expression of HMGB1 in unilateral IR sections. Unlike its distribution in AKI sections, HMGB1 was mainly detected in extracellular areas adjacent to the damaged cells and in the cytoplasm of damaged cells (Figure 7A), indicating HMGB1 passive release following IR insult. However, the passive release was attenuated by GSPE (Figure 7A). Western blot analysis further supported this result (Figure 7B,C).

**Figure 8:**
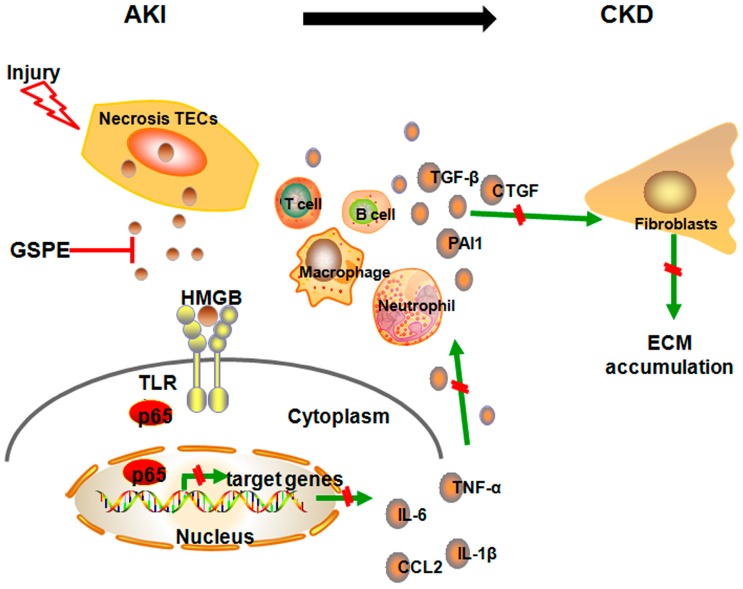
Schematic diagrams illustrating the mechanism by which GSPE protects against IR-induced renal insult by inhibiting the HMGB1-TLR4-p65 pathway. Once tubular epithelial cells suffer IR-induced damage and necrosis, HMGB1 is transported from the nucleus to cytoplasm and released into the extracellular milieu, where they can then bind to TLR4 and promote p65 translocation to the nucleus through autocrine or paracrine signaling. However, all of those effects can be lessened by GSPE. Decreased NF-κB p65 transcription and translation led to less cytokine and chemokine release, which can trigger a pro-inflammatory response and activate immune cells. Decreased tubulointerstitial inflammation can then inhibit the activation of fibroblasts and mitigate the tubular damage. At the same time, successive treatment with GSPE can also inhibit the HMGB1-TLR4-p65 pathway, which leads to not only less inflammation but also the release of the most potent pro-fibrosis cytokine TGF-β. Green arrows mean promotion and red line means inhibition.

In line with the previous experiments, we next measured the HMGB1 receptors, TLR4, TLR2 and RAGE, and the effector protein, nuclear p65. The same trends were observed in unilateral sections as in AKI. GSPE mitigated the upregulation of TLR4 and nuclear p65 (Figure 7D–F), which had been seen in acute IR insult sections (Figure 7D–F), but had no effect on TLR2 and RAGE (Figure 7D). Because HMGB1 has been found to participate in the release of TGF-β by renal tubular epithelial cells [16,17] and because TGF-β is the most prominent pro-fibrosis cytokine in the development of renal fibrosis, its expression in the kidneys was assessed by Western blot. After 14 days of unilateral IR, kidney tissues from mice treated with GSPE showed much lower TGF-β expression compared with that of PBS treated animals (Figure 7G). At last, we used a schematic diagram to illustrate the mechanism by which GSPE protects against IR-induced renal insult by inhibiting the HMGB1-TLR4-p65 pathway (Figure 8).

## 3. Discussion

In this study, we first established an acute renal injury model, bilateral I/R, and a chronic renal fibrosis model, unilateral I/R, which imitated the AKI and subsequent CKD, respectively, and administered GSPE to both models. The results showed that GSPE can not only attenuate acute renal tubular damage, accompanied by a remarkable reduction in the renal functional impairment as assessed by BUN, SCr and KIM-1, but can also mitigate tubulointerstitial fibrosis. The protective effect of GSPE seems to be related to its prevention of the nucleocytoplasmic translocation and release of HMGB1.

GSPE is an efficient natural antioxidant extracted from grape seeds. Previous studies have shown the renoprotective effects of GSPE in acute renal injury, mainly focused on its ability to remove reactive oxygen species (ROS) or inhibit endoplasmic reticulum (ER) stress-induced apoptosis [23,24,25]. However, GSPE also exhibits anti-inflammatory properties in many other diseases [26,27,28]. Thus, in the present study, we pretreated C57BL/6C mice with three successive doses of GSPE for 5 days before bilateral I/R and measured its anti-inflammatory effects. Impressively, GSPE administered at 250 mg/kg maximally reduced tubular necrosis, endothelial injury, renal cell apoptosis, neutrophil and macrophage infiltration, and inflammatory cytokine and chemokine production, all of which are seen in the PBS treatment group. It was interesting to note that the protective effects of GSPE were not dose-dependent and that a higher concentration did not improve renoprotection.

GSPE can notably attenuate the inflammatory reaction in acute I/R, and inflammation is an important risk factor contributing to the development of chronic kidney disease [7,29]. We tried to administer GSPE for 14 days after unilateral I/R. In contrast to the case with acute I/R, 150 mg/kg GSPE had the best anti-fibrotic effect. At the same time, GSPE also attenuated tubular injury, macrophage infiltration, and the secretion of potent cytokines and chemokines, such as IL-6, IL-1β, TNF-α, and CCL2. In all cases, our results demonstrated, for the first time, that GSPE can have a potent protective effect on both bilateral I/R and unilateral I/R. It was therefore important to address the molecular mechanisms by which GSPE exerted its renoprotection in these two models.

HMGB1 is a nuclear protein that stabilizes nucleosomes and allows the bending of DNA that facilitates gene transcription. Recently, however, it has been studied extensively as a DAMP molecule that can be passively released by necrotic cells. Moreover, as a potent innate alarm signal, HMGB1 alerts the innate immune system to initiate host defense against invading pathogens or the reparative response in case of tissue or organ injury. In addition, extracellular HMGB1 can also enhance chemotactic and adaptive immune responses through paracrine and autocrine activity [30]. A great many animal models have demonstrated that HMGB1 is an early mediator of injury and inflammation in IRI [31,32], and that neutralizing HMGB1 can significantly inhibit the inflammatory response and decrease renal damage after I/R [15]. In view of this, we measured the expression of HMGB1 in both two models, as it mainly exhibits nucleocytoplasmic translocation in acute I/R but is released into the extracellular milieu in chronic fibrosis, and we found that both were attenuated by GSPE.

HMGB1 exerts its proinflammatory effect by interacting with pattern recognition receptors, such as TLR2, TLR4 and RAGE [13,33], so we measured the mRNA of these three genes by real-time PCR. GSPE can apparently inhibit increases in TLR4, but not RAGE and TLR2, including protein expression. In line with this notion, we found that the expression of nuclear P65, which transcribes inflammatory cytokines relevant to renal injury and fibrosis, was also mitigated by GSPE. In addition, given that TGF-β is the most potent pro-fibrotic cytokine, we measured the protein expression of TGF-β in unilateral I/R model; surprisingly, the TGF-β level was also lowered by GSPE.

In conclusion, we have provided evidence demonstrating that pretreatment with GSPE can provide protection against IR-induced acute renal injury by inhibiting the HMGB1-TLR4-P65 pathway and the downstream inflammatory reaction and that consecutively treatment with GSPE after I/R can reduce tubulointerstitial inflammation; thus, renal fibrosis was also decreased. Together, our data supports the idea that administering GSPE prior or after to IR insults could be a viable approach to prevent IR-induced acute renal injury and chronic fibrosis in clinical settings.

## 4. Experimental Section

### 4.1. Animals

Male C57BL/6 mice (8 weeks old, 25–30 g) were purchased from Hua Fukang Experimental Animal Center (Beijing, China) and maintained under specific pathogen-free (SPF) conditions. After a minimum of 7 days of acclimation, the mice were randomly divided into six groups, with each containing 7 mice: (a) the I/R-vehicle group (IRI+PBS), in which mice were administered PBS and subjected to bilateral or unilateral renal ischemia for 30 min; (b) the I/R-GSPE group (IRI+GSPE), in which mice were pre-treated with 150, 250 or 500 mg/kg of GSPE orally for 5 days before bilateral IR insult or successive administration of GSPE for 14 days after unilateral IR; (c) the sham group (Sham), in which mice underwent similar surgical procedures but without renal ischemia and reperfusion; and (d) the GSPE group (GSPE), in which mice were administered GSPE and the other treatments were the same as those in the sham group. GSPE contained ≥95.0% proanthocyanidins and was purchased from Jianfeng, Inc. (Tianjin, China). This study was approved by Guidelines for Experimental Animal Ethical Committee of Huazhong University of Science and Technology (No.: TJ-A20150502, 2 May 2015). All procedures were approved and performed in accordance with the institutional guidelines for animal care.

### 4.2. Renal IRI Model

First, animals were anaesthetized with a 1% sodium pentobarbital solution (6 mL/kg) by intraperitoneal injection and then placed in a prone position to maintain their body temperature at 36.8–37.2 °C during surgery. After a bilateral or unilateral dorsal incision to expose the kidneys, the renal artery was occluded using a microvascular clamp (RoBoz Surgical Instrument Co., Gaithersburg, MD, USA) for 30 min. After that, clamps were removed and the animals were allowed to recover with free access to food and water. The mice subjected to bilateral IR were sacrificed 24 h after reperfusion, and unilateral IR mice were sacrificed 14 days after reperfusion. Plasma samples and kidneys were collected for further experiments.

### 4.3. Assessment of Renal Function

Blood samples were obtained from the inferior vena cava 24 h after reperfusion. Blood urea nitrogen (BUN) and serum creatinine (Cr) levels were assayed at the Department of Clinical Laboratories of Tongji Hospital to assess renal function.

### 4.4. Histological Analysis

Paraffin-embedded renal sections (4 μm) were subjected to PAS, Masson′s, and Sirius Red staining as previously reported [34]. Morphological changes in the cortex and medulla were scored by two pathologists in a blinded fashion using a semi-quantitative design to evaluate the degree of tubular necrosis, the integrity of the brush border and the basement membrane, and the cast formation on a five-point scale, based on the area of involvement of the injury as follows: 0, <10%; 1, 10%–25%; 2, 25%–50%; 3, 50%–75% and 4, 75%–100% [35]. Tubulointerstitial fibrosis was analyzed according to the percentage of fibrosis in the tubulointerstitial area using the Image Pro Plus software (Media Cybernetics, Rockville, MD, USA) in >10 random fields [34].

### 4.5. Immunohistochemical Staining 

Paraffin-embedded renal sections (4 μm) were deparaffinized in xylene and rehydrated in graded alcohol. First, endogenous peroxidase was blocked with 3% H_2_O_2_, and non-specific proteins were blocked with 10% goat serum for 30 min. Then, the sections were incubated with antibodies against HMGB1, collagen IV, PDGFR-β, α-SMA (Abcam, Cambrige, MA, USA) and MPO (Thermo scientific, Waltham, MA, USA) at 4 °C overnight, followed by incubation with an HRP-conjugated secondary antibody at room temperature for 30 min. After incubation with the chromogenic substrate (DAB), the sections were counterstained with hematoxylin. The intensity was assessed in a blinded manner by randomly selecting 10 fields per section at a magnification of 400×, and 7 mice were examined in each study group.

### 4.6. Immunofluorescence

Paraffin-embedded sections (4 μm) were deparaffinized as described above and blocked with 10% goat serum for 30 min. They were then incubated with antibodies against F4/80 (Santa Cruz Biotechnology, Santa Cruz, CA, USA) or LY6G (Abnova, San Diego, CA, USA) at 37 °C for 2 h and subsequently visualized using a FITC-conjugated secondary antibody. The nucleus was counterstained with DAPI. Color images were obtained under a Nikon fluorescence microscope (Nikon ECLIPSE TE2000-U, Tokyo, Japan).

### 4.7. Apoptosis Assay

Apoptosis was determined by a terminal deoxynucleotidyl transferase-mediated uridine triphosphate nick end labeling (TUNEL) assay with a kit from Roche Diagnostics (Indianapolis, IN, USA) using established techniques [30]. The number of apoptotic cells was counted in 10 high-power (400×) fields for quantification.

### 4.8. Isolation of the Total, Cytoplasmic and Nuclear Proteins

Total renal protein was isolated with RIPA lysis buffer containing 1 mM phenylmethylsulfonyl fluoride (Amresco, Solon, OH, USA) and a protease inhibitor cocktail (Roche, Indianapolis, IN, USA). The kidney was cut into pieces and suspended in cell lysis buffer and then lysed by homogenization at 4 °C. After centrifugation at 12,000 rpm for 10 min, the total protein in the supernatants was collected. The cytoplasmic and nuclear proteins were isolated by the Nuclear and Cytoplasmic Protein Extraction Kit (Beyotime Institute of Biotechnology, Shanghai, China). Fresh renal tissues were suspended in a buffer containing the cytoplasmic protein extraction reagent and lysed by homogenization for 30 min on ice. After centrifugation at 12,000× *g* for 5 min, the cytoplasmic proteins in the supernatants were collected. The sediment was then suspended in nuclear protein extraction buffer, vortexed repeatedly for 30 min on ice and centrifuged at 12,000× *g* for 5 min, and then, the nuclear proteins were collected. All of the proteins were stored at −80 °C for Westernblot analysis.

### 4.9. Western Blot

Fifty micrograms of proteins was separated by 12% SDS-PAGE and then electrophoretically transferred onto PVDF membranes. After blocking with 5% non-fat milk 1 h at room temperature, the membranes were incubated with antibodies in optimal dilutions against HMGB1, TGF-β, collagen I, PDGFR-β, α-SMA (Abcam, Cambrige, MA, USA), TLR4 (Santa Cruz Technologies, Santa Cruz, CA, USA) or p65 (Cell Signaling Technology, Danvers, MA, USA) at 4 °C overnight and then incubated with an HRP-conjugated secondary antibody. The target bands were detected using the ECL Plus Western blot kit (PIERCE, Rockford, IL, USA). The density of the bands was quantified using Labworks image acquisition and analysis software (UVP, Fremont, CA, USA).

### 4.10. Real-Time PCR

Total RNA was isolated from renal tissues using TRIzol reagent (Invitrogen, Shanghai, China) according to the manufacturer’s instructions, and 2 μg of total RNA was reverse transcribed into cDNA using the Reverted First Stand cDNA Synthesis Kit (Thermo Scientific, Waltham, MA, USA). Real-time PCR was carried out using the LightCycler 480 system (Roche, Pleasanton, CA, USA) with the primers shown in Table 1. The relative amounts of mRNA were normalized to β-actin and calculated using the 2^−∆∆*C*t^ approach as previously reported [36].

### 4.11. Statistical Analyses

All data are expressed as the mean ±SEM. The Graphpad Prism 5 software (GraphPad Software, La Jolla, CA, USA) was used for statistical analysis, using Student’s t-test or one-way or two-way ANOVA where appropriate. A *p* value <0.05 was considered significant.

## Figures and Tables

**Table 1:** Primers used to amplify cDNAs for mice.

Primers	Sequence (Sense/Antisense)
Kim-1	5′-TAAACCAGAGATTCCCACAC-3′
	5′-GATCTTGTTGAAATAGTCGTGG-3′
IL-6	5′-TAGTCCTTCCTACCCCAATTTCC-3′
	5′-TTGGTCCTTAGCCACTCCTTC-3′
TNF-α	5′-CCTGTAGCCCACGTCGTAG-3′
	5′-GGGAGTAGACAAGGTACAACCC-3′
IL-1β	5′-GAAATGCCACCTTTTGACAGTG-3′
	5′-TGGATGCTCTCATCAGGACAG-3′
CCL2	5′-TAAAAACCTGGATCGGAACCAAA-3′
	5′-GCATTAGCTTCAGATTTACGGGT-3′
CCL3	5′-TGTACCATGACACTCTGCAAC-3′
	5′-CAACGATGAATTGGCGTGGAA-3′
CCL5	5′-GCTGCTTTGCCTACCTCTCC-3′
	5′-TCGAGTGACAAACACGACTGC-3′
CXCL1	5′-ACTGCACCCAAACCGAAGTC-3′
	5′-TGGGGACACCTTTTAGCATCTT-3′
CXCL5	5′-TGCGTTGTGTTTGCTTAACCG-3′
	5′-CTTCCACCGTAGGGCACTG-3′
ICAM-1	5′-GTGATGCTCAGGTATCCATCCA-3′
	5′-CACAGTTCTCAAAGCACAGCG-3′
TLR4	5′-GCCTTTCAGGGAATTAAGCTCC-3′
	5′-GATCAACCGATGGACGTGTAAA-3′
TLR2	5′-TCTAAAGTCGATCCGCGACAT-3′
	5′-CTACGGGCAGTGGTGAAAACT-3′
RAGE	5′-ACTACCGAGTCCGAGTCTACC-3′
	5′-CCCACCTTATTAGGGACACTGG-3′
β-actin	5′-GGCTGTATTCCCCTCCATCG-3′
	5′-CCAGTTGGTAACAATGCCATGT-3′
